# Prenatal Maternal Stress, Socioeconomic and Cultural Determinants, and Early Childhood Physical and Neurodevelopmental Outcomes: Evidence from a Prospective Cohort Study in Georgia

**DOI:** 10.3390/healthcare14152247

**Published:** 2026-07-23

**Authors:** Nia Masiukovichi, William Michael Caudle, Tamari Masiukovichi, Elza Nikoleishvili

**Affiliations:** 1 School of Health Sciences, University of Georgia, Tbilisi 0177, Georgia; e.nikoleishvili@ug.edu.ge; 2 Gangarosa Department of Environmental Health, Rollins School of Public Health, Emory University, Atlanta, GA 30322, USA; william.m.caudle@emory.edu; 3 Department of Pharmaceutical, Toxicological and Medicinal Chemistry, Tbilisi State Medical University, Tbilisi 0186, Georgia; tatia.masiukovich@yahoo.com

**Keywords:** prenatal stress, early childhood development, neurodevelopmental outcomes, prospective cohort study, Georgia

## Abstract

**Background:** Prenatal maternal stress is increasingly recognized as a critical determinant of fetal programming, with potential consequences for early physical growth and neurodevelopment. Although a growing body of evidence links maternal psychological stress to adverse perinatal and developmental outcomes, most studies originate from high-income Western settings, rely predominantly on self-reported stress measures, and rarely incorporate biological stress markers. Evidence from post-Soviet regional contexts remains limited. **Methods:** A two-phase study was conducted in Tbilisi, Georgia. Phase I employed a cross-sectional observational design involving 398 pregnant women recruited from maternity homes and women’s consultations. Maternal psycho-emotional stress was assessed using a study-specific pregnancy stress questionnaire, and salivary cortisol was measured in a subsample of women reporting chronic stress (N = 95). Phase II used a prospective cohort design to follow infants born to stress-exposed mothers (N = 95) and non-stressed controls (N = 95) from birth to 12 months. Infant outcomes included anthropometric measurements evaluated using World Health Organization Child Growth Standards and neurodevelopmental screening using the Ages & Stages Questionnaires, Third Edition (ASQ-3), at 1–3, 5–7, and 11–13 months. Statistical analyses were made by SPSS 23.0. **Results:** Salivary cortisol assessment in the stress-exposed subsample provided supportive biological evidence of elevated stress levels but was not used for direct comparison with the control group. Children born to stress-exposed mothers showed significantly poorer physical growth and lower ASQ-3 scores across all developmental domains during the first year of life (all *p* < 0.05). **Conclusions:** This study provides novel evidence from an underrepresented regional context and supports early developmental surveillance and preventive strategies to improve maternal and child health outcomes.

## 1. Introduction

The intrauterine environment constitutes the earliest setting in which biological and psychosocial exposures influence lifelong health. Within the Developmental Origins of Health and Disease (DOHaD) framework, prenatal conditions are understood to shape later cardiovascular, metabolic, immune, and neurodevelopmental outcomes through fetal programming mechanisms [1].

Among prenatal exposures, maternal psychosocial stress has emerged as an important and potentially modifiable determinant of child health. Prenatal stress, defined as perceived exposure to unpredictable or uncontrollable adversity and encompassing anxiety, depressive symptoms, and emotional dysregulation [2,3], varies in timing, intensity, and chronicity, all of which may differentially affect fetal development [4,5]. Maternal stress is also socially patterned. Socioeconomic disadvantage, lower educational attainment, occupational strain, limited access to healthcare, and cultural context strongly influence stress exposure, placing prenatal stress within the broader framework of social determinants of health [6,7].

A substantial body of evidence links elevated maternal stress during pregnancy to adverse offspring outcomes. Associations have been reported with neurodevelopmental and neuropsychiatric conditions, including autism spectrum disorder, attention-deficit/hyperactivity disorder, anxiety, and depression [2,4,5,6]. Beyond mental health outcomes, prenatal stress has also been associated with altered stress physiology, immune function, autonomic regulation, and increased childhood morbidity [7,8,9]. Emerging evidence further suggests that stress-related neurobiological differences may already be detectable during fetal life, including alterations in brain structure and functional connectivity [10,11]. These findings underscore the prenatal period as a foundational stage in developmental trajectories and a critical window for early prevention.

From a global health perspective, reducing prenatal stress is closely aligned with the World Health Organization’s Sustainable Development Goals, particularly SDG 3, which prioritizes maternal, newborn, and child health, as well as early childhood development [12,13,14]. Addressing prenatal stress may therefore contribute not only to improved physical and cognitive outcomes in children, but also to the reduction of intergenerational cycles of health inequity.

Evidence regarding prenatal maternal stress and its long-term effects on offspring remains limited in the South Caucasus region. Georgia represents a unique socio-cultural setting characterized by ongoing socioeconomic transitions, distinct family and social support structures, and healthcare system challenges that may influence both the prevalence and sources of maternal stress, potentially differing from those reported in Western populations.

## 2. Materials and Methods

### 2.1. Study Design and Setting

This study examined the association between maternal psycho-emotional stress, salivary cortisol levels during pregnancy, and early physical and cognitive development in children up to 12 months of age.

The study was conducted in Tbilisi, Georgia, in maternity hospitals and women’s consultation centers providing routine obstetric care. Postnatal follow-up assessments were carried out during scheduled pediatric visits at outpatient clinics and polyclinics.

A two-phase design was used, combining a cross-sectional assessment of prenatal maternal stress with a prospective cohort follow-up of offspring to evaluate associations between prenatal psycho-emotional stress, salivary cortisol concentrations, and early childhood physical and cognitive outcomes.

Phase I consisted of a cross-sectional evaluation of maternal psycho-emotional stress and related socioeconomic, environmental, and cultural factors during pregnancy. Phase II followed mother–infant dyads from birth to 12 months to assess infant growth and neurodevelopment.

Data were collected from April 2023 to August 2025.

### 2.2. Ethical Considerations

The study protocol and all instruments were approved by the Ethics Committee of the University of Georgia (Approval No. UGREC-03-23). Written informed consent was obtained from all participants before enrollment. Parental consent covered all infant assessments. Data were anonymized, and participants were informed of their right to withdraw at any time without consequences.

### 2.3. Sample Size Calculation

The minimum required sample size was estimated using the average annual number of live births in Georgia (2016–2020) as the source population (N). Cochran’s formula for a single proportion was applied assuming:

- 95% confidence level (Z = 1.96);
- 5% margin of error (d = 0.05);
- Conservative prevalence estimate (*p* = 0.50).

The formula used was: n_0_ = (Z^2^ × *p* × (1 − *p*))/d^2^

A finite population correction was subsequently applied:

n = n_0_/[1 + (n_0_ − 1)/N]

The final minimum required sample size was **N = 398**.

### 2.4. Participants

**Inclusion Criteria**

Pregnant women were eligible if they:

- Were aged ≥ 18 years;
- Were in the first, second, or third trimester;
- Were residents of Tbilisi, Georgia;
- Had no pre-existing severe chronic medical conditions or diagnosed psychological disorders;
- Were receiving regular prenatal care;
- Provided written informed consent and agreed to complete questionnaires and provide saliva samples.

**Exclusion Criteria**

Participants were excluded if they:

- Were aged > 40 years;
- Had high-risk pregnancies (e.g., multiple gestation, preeclampsia, gestational diabetes, placenta previa);
- Had chronic systemic diseases (e.g., cardiovascular, renal, autoimmune disorders);
- Had severe untreated psychiatric disorders;
- Were using psychotropic or teratogenic medications;
- Were enrolled in similar pregnancy-related intervention studies;
- Had significant language barriers preventing informed consent;
- Carried fetuses with known genetic or congenital abnormalities;
- Were unable to provide informed consent.

### 2.5. Assessment of Maternal Stress (Phase I)

Maternal psycho-emotional stress was assessed using a study-specific 46-item questionnaire consisting of multiple-choice and open-ended items.

The questionnaire evaluated:

- Emotional well-being (anxiety, depressive symptoms);
- Social support;
- Educational attainment;
- Socioeconomic conditions;
- Material status;
- Cultural influences;
- Family circumstances.

For Phase II of the study, a subgroup of participants was randomly selected from the Phase I cohort. Specifically, 95 women from the stress group (n = 172) and 95 women from the control group (n = 226) were randomly chosen for longitudinal follow-up and additional assessments. No matching procedure was applied, and participant selection was not based on demographic, socioeconomic, obstetric, or other clinical characteristics.

A subgroup of women reporting chronic continuous stress (N = 95) provided saliva samples for cortisol measurement.

### 2.6. Salivary Cortisol Measurement

To provide a biological plausibility of stress, afternoon salivary cortisol samples were collected from the stress-exposed subgroup (N = 95).

Salivary cortisol measurements were performed in a randomly selected subgroup of participants from the stress group (N = 95). The purpose of cortisol assessment was to provide an objective physiological indicator supporting the presence of stress among women who had already been classified as experiencing prenatal stress based on validated psychological assessment methods. Cortisol measurements were not obtained from the control group, as the analysis was intended to characterize the biological stress response within the stress-exposed subgroup rather than to perform direct biological comparisons between stressed and non-stressed participants.

Participants were instructed to refrain from eating, drinking, or brushing teeth for at least 30 min prior to sampling.

Samples were stored at +4 °C and analyzed using an enzyme-linked immunosorbent assay (ELISA) (Cortisol ELISA Kit, m). Optical density was measured at 405 nm.

Cortisol measurement was used to support the biological plausibility of stress exposure but was not used as the sole criterion for exposure classification.

### 2.7. Child Physical Growth Assessment (Phase II)

Postnatal anthropometric assessments were conducted in:

- Infants of stress-exposed mothers (N = 95);
- Infants of non-stressed controls (N = 95).

Standardized measurements included:

- Body length/height;
- Body weight;
- Head circumference;
- Chest circumference.

Measurements were obtained using calibrated instruments and evaluated according to WHO Child Growth Standards (WHO, 2006) [15].

### 2.8. Neurodevelopmental Assessment

Neurodevelopment was assessed at:

- 1–3 months;
- 5–7 months;
- 11–13 months.

The Ages & Stages Questionnaires, Third Edition (ASQ-3), was used. The instrument evaluates five domains:

- Communication;
- Gross motor;
- Fine motor;
- Problem-solving;
- Personal–social development.

Questionnaires were completed by parents during routine pediatric visits with standardized guidance from trained pediatricians.

### 2.9. Translation and Cultural Adaptation of the ASQ-3

As an officially validated Georgian version was unavailable, the ASQ-3 was translated using standardized cross-cultural procedures:

1. Two independent forward translations (English → Georgian);
2. Blinded back-translation (Georgian → English);
3. Reconciliation by expert panel;
4. Pilot testing among parents (N = 12);
5. Minor linguistic refinements.

### 2.10. Statistical Analysis

Statistical analyses were performed using SPSS software (version 23.0; IBM Corp., Armonk, NY, USA). Continuous variables are presented as means ± standard deviations (SD), while categorical variables are presented as frequencies and percentages. Differences between groups for continuous variables were assessed using Student’s *t*-test. Associations between categorical variables were evaluated using Pearson’s Chi-square test. Logistic regression analysis was used to calculate odds ratios (ORs) and 95% confidence intervals (CIs) for factors associated with prenatal stress and study outcomes.

A two-sided *p*-value of <0.05 was considered statistically significant.

## 3. Results

### 3.1. Participant Characteristics

A total of 398 pregnant women were enrolled in the study, including 172 women in the prenatal stress group (Study group or the Group 1) and 226 in the control group (Group 2). Baseline characteristics of the study participants are demonstrated in Table 1.

Prenatal stress was confirmed using validated self-assessment questionnaires and, in a subsample (n = 95), salivary cortisol measurements. Saliva cortisol levels are presented in Supplementary Figure S6.

The two groups did not differ significantly in maternal age (t = −1.72, *p* = 0.086), educational attainment (χ^2^ = 0.66, *p* = 0.719), or dwelling location (χ^2^ = 2.91, *p* = 0.088), indicating baseline comparability in key demographic characteristics.

### 3.2. Socioeconomic and Cultural Factors Associated with Prenatal Stress

The factors associated with stress during pregnancy are demonstrated in Supplementary Figure S1 and Supplementary Table S1.

Significant associations were observed between prenatal stress and selected socio-economic and cultural factors (Supplementary Table S4).

Pregnant women who continued their usual workload during pregnancy had higher odds of belonging to the stress group compared with those not working or on maternity leave (OR = 1.82, 95% CI: 1.10–3.02, *p* = 0.019). Overtime work during pregnancy demonstrated a particularly strong association with prenatal stress (OR = 3.05, *p* = 0.001).

Household structure was also associated with stress exposure. Women living in smaller households (1–2 cohabitants) had increased odds of prenatal stress compared with those living in larger family units (OR = 1.59, 95% CI: 1.02–2.45, *p* = 0.038). Additionally, absence of a partner was significantly more frequent in the stress group (*p* = 0.010).

Reproductive history differed between the groups. A prior spontaneous abortion was significantly more common among women with prenatal stress (OR = 2.19, 95% CI: 1.37–3.49, *p* = 0.001), as was a history of artificial abortion (OR = 2.10, 95% CI: 1.22–3.64, *p* = 0.007).

No statistically significant differences were observed between groups with respect to monthly income, housing space per person, marriage registration type, free antenatal patronage, or regular gynecological visits.

### 3.3. Early Childhood Physical Growth Outcomes

A total of 190 children (95 per group) were followed longitudinally from birth to 12 months of age.

### 3.4. Birth Outcomes

The odds of delivery complications were nearly three times higher in the study group (OR = 2.98, 95% CI: 1.55–5.70; *p* = 0.001), indicating a statistically significant association. The study group had significantly higher odds of weakness of delivery activities (OR = 2.21, 95% CI: 1.13–4.33; *p* = 0.021) (Supplementary Figures S2 and S3).

Mean newborn chest circumference was slightly lower in the study group than in the control group (31.5 ± 1.8 cm vs. 32.2 ± 1.7 cm. *p* = 0.012).

Low 1 min APGAR score was observed in 38.9% of the study group compared with 23.1% of controls (OR = 2.12, *p* = 0.028) (Supplementary Figure S4).

No statistically significant differences between groups were identified in birth weight, birth length, head circumference, or 5 min APGAR score (*p* > 0.01).

### 3.5. Postnatal Growth Trajectories

Differences in body weight was observed after birth throughout the infancy. At the mean age of 2, 6, and 12 months postpartum, children in the stress group had significantly lower mean body weight compared with the controls (*p* < 0.001) (Supplementary Tables S8 and S9).

Similarly, mean body height and head circumference remained significantly lower at each age-group assessment (*p* < 0.001) (Supplementary Tables S10 and S11) although both groups demonstrated expected physiological growth over time.

Low chest circumference was significantly more prevalent in the stress group at 1–3 months postpartum (*p* = 0.046), but intergroup differences were not consistently observed at later time points. The data of children’s chest circumference in different age-groups in the study and control groups are presented in Supplementary Table S12.

Overall, these findings indicate sustained growth disparities during the first year of life among the children exposed to prenatal maternal stress. The anthropometric outcomes across the first year of life, and the gain of these parameters in dynamic and their comparison between Study and Control groups, are demonstrated in Supplementary Tables S5 and S6.

### 3.6. Early Cognitive Developmental Outcomes

Neurodevelopmental outcomes were assessed using the Ages & Stages Questionnaire, Third Edition (ASQ-3), at the mean age of 2, 6, and 12 months postpartum.

### 3.7. Communication Domain

**Figure 1:**
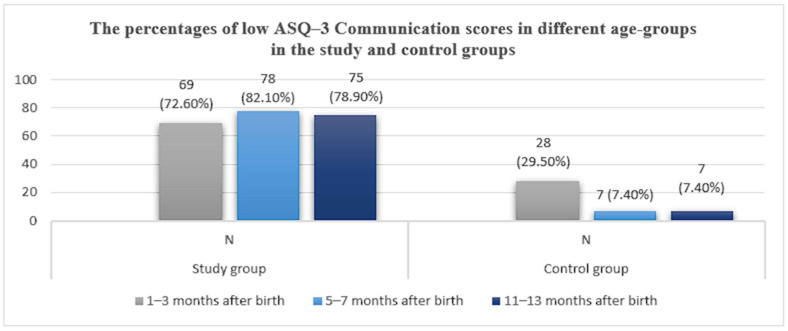
Shows that the proportion of infants with low ASQ-3 communication scores was consistently higher in the study group than in the control group across all postnatal assessment periods. In the study group, low communication scores were observed in 72.6% of infants at 1–3 months, 82.1% at 5–7 months, and 78.9% at 11–13 months. In contrast, the corresponding proportions in the control group were substantially lower: 29.5%, 7.4%, and 7.4%, respectively. Overall, the figure demonstrates a persistent excess of communication delay in the study group throughout the first year of life.

At 2 months postpartum, a significantly higher proportion of infants in the stress group demonstrated low communication scores compared with controls (72.60% vs. 29.50%, *p* < 0.001). Elevated prevalence persisted at 6 and 12 months (both *p* < 0.001) (Figure 1).

### 3.8. Gross and Fine Motor Development

Low gross motor scores were significantly more frequent in the stress group at 2 months (53.7% vs. 29.5%, *p* < 0.001) and remained elevated at 6 and 12 months (both *p* < 0.001). Similarly, low fine motor scores were substantially more prevalent in the stress group at all follow-up points (all *p* < 0.001). The results are shown in Supplementary Tables S2 and S3.

### 3.9. Problem-Solving and Personal–Social Domains

**Figure 2:**
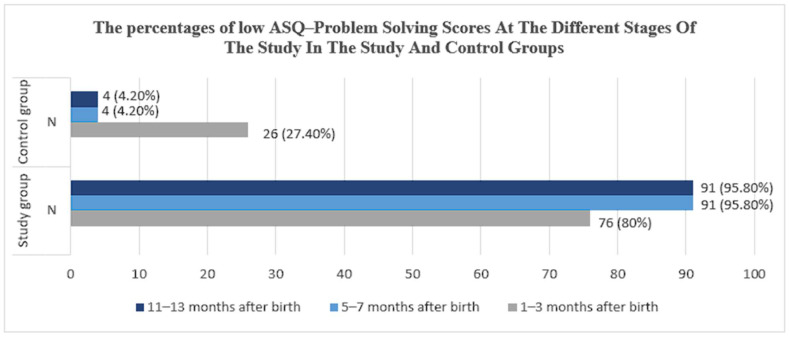
Demonstrates marked between-group differences in the prevalence of low ASQ problem-solving scores across the first year of life. In the study group, the proportion of infants with low problem-solving scores was already high at 1–3 months (80.0%) and increased further at 5–7 months and 11–13 months (95.8% at both time points). In contrast, the corresponding prevalence in the control group was substantially lower, decreasing from 27.4% at 1–3 months to 4.2% at both later assessments. Overall, the figure indicates a persistent and pronounced excess of problem-solving delay in the study group throughout infancy.

**Figure 3:**
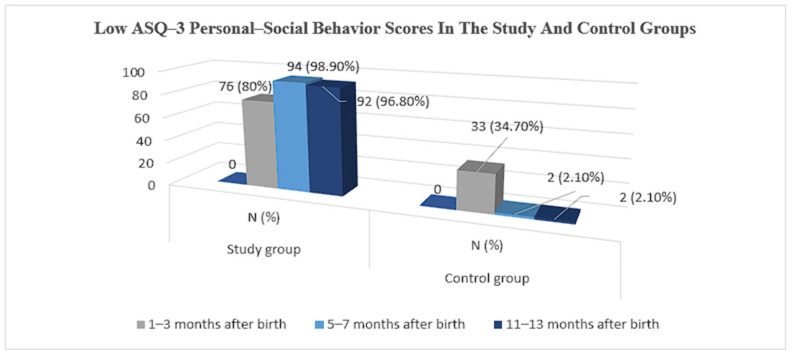
Shows marked between-group differences in low ASQ-3 personal–social scores across infancy. In the study group, no infants had low scores at birth, but the prevalence increased sharply to 80.0% at 1–3 months, 98.9% at 5–7 months, and remained very high at 96.8% at 11–13 months. In contrast, the control group showed substantially lower proportions, rising to 34.7% at 1–3 months and declining to 2.1% at both later assessments. Overall, the figure demonstrates a pronounced and persistent excess of personal–social developmental delay in the study group during the first year of life.

Infants in the stress group demonstrated significantly higher prevalence of low problem-solving scores at 2, 6, and 12 months (all *p* < 0.001). Personal–social developmental delays were also more frequent in the stress group across all time points (all *p* < 0.001) (Figure 2 and Figure 3).

Overall, the prevalence of developmental delays/low scores across the first year of life, according to 5 domains of ASQ-3 and the comparison of all the domains between the study and control groups is presented in Supplementary Table S7.

### 3.10. Anxiety-Related Problems

While no significant differences in behavioral problems were observed in early infancy, ASQ-3 detected anxiety-related problems were significantly more prevalent in the study group at 6 and 12 months postpartum, compared to control group (*p* < 0.001) (Supplementary Figure S5).

### 3.11. Socioeconomic Moderators of Child Outcomes

Analyses of maternal education and occupational status demonstrated time-specific associations with selected anthropometric and emotional outcomes. However, no consistent associations were observed across cognitive domains of the offspring.

Collectively, these findings suggest that prenatal stress is independently associated with early growth restriction and elevated risk of developmental delay across multiple domains during the first year of life. Socioeconomic and relational stressors appear to contribute significantly to maternal stress exposure and may indirectly influence early childhood outcomes.

## 4. Discussion

This study found that prenatal maternal stress was significantly associated with less favorable physical growth and neurodevelopment during the first year of life. Children born to stress-exposed mothers had poorer perinatal indicators, including more frequent delivery complications, weakness of labor activity, and low 1 min APGAR scores, followed by persistently lower anthropometric measures and markedly higher frequencies of low ASQ-3 scores across all assessed developmental domains. These findings are consistent with the Developmental Origins of Health and Disease (DOHaD) framework, which proposes that prenatal exposures may shape long-term biological and developmental trajectories [2,16].

The study also showed that prenatal stress was shaped less by broad demographic factors than by occupational burden, social support, and reproductive history, and that these stress exposures had measurable consequences for infant growth and neurodevelopment [16,17]. Maternal stress during pregnancy may influence fetal development through activation of the hypothalamic–pituitary–adrenal axis, altered glucocorticoid exposure, and downstream neuroendocrine effects on fetal growth and brain development [2,8,9,16]. In our cohort, elevated salivary cortisol levels among women in the stress group supported concordance between self-reported stress and a biological marker of stress, strengthening the interpretation that prenatal stress was not only psychosocially reported but also physiologically reflected. This finding aligns with previous evidence that maternal stress physiology during pregnancy may become biologically embedded and influence infant stress regulation and later developmental outcomes [7,8].

A major finding of the present study is that children in the prenatal stress group demonstrated poorer physical growth not only at birth but throughout infancy. Although both groups showed expected physiological growth over time, body weight, body length, head circumference, and chest circumference remained consistently lower in the exposed group across follow-up assessments. These findings support previous reports linking prenatal stress with altered fetal growth and later developmental vulnerability [2,9,16].

The neurodevelopmental results were particularly striking. Infants in the stress-exposed group had significantly higher frequencies of low ASQ-3 scores in communication, gross motor, fine motor, problem-solving, and personal–social domains at each follow-up point, and anxiety-related concerns emerged more frequently later in infancy. This broad pattern is consistent with previous literature linking prenatal maternal distress with altered infant stress regulation, emotional development, and later vulnerability to anxiety, behavioural problems, and neuropsychiatric outcomes [4,5,6,7,9,18]. More recent evidence suggests that maternal stress during pregnancy may also be associated with altered fetal brain growth, cortical maturation, and functional neurodevelopment during intrauterine life [10,11]. Our findings extend this literature by demonstrating that developmental vulnerability may be detectable across multiple domains during the first year of life in a Georgian cohort.

The most consistent group differences were observed in communication and problem-solving domains. This interpretation is supported by evidence linking maternal stress during pregnancy with alterations in fetal and early brain development, including neural systems involved in cognition, affect regulation, and later learning [10,17]. Marked deficits in personal–social functioning further suggest that prenatal stress may affect infant socioemotional development through both biological and postnatal caregiving pathways. Maternal psychological distress may influence early child outcomes not only through fetal programming but also through its effects on caregiver responsiveness, emotional availability, and the early relational environment [19,20,21].

Another important contribution of this study is the demonstration that prenatal stress was socially patterned. Stress exposure was significantly associated with continuation of usual work during pregnancy, overtime work, smaller household size, absence of a partner, and prior spontaneous or induced abortion, whereas age, education, income, place of residence, and access to prenatal care were not associated with group membership. These findings support the view that prenatal stress should be understood not only as an individual psychological state, but also as a socially embedded exposure shaped by occupational burden, reproductive history, and support structures [2,3,18]. They further reinforce the argument that maternal stress belongs within the broader framework of social determinants of health and should be addressed through clinical and public health strategies [13,14,19,21].

The absence of significant associations with maternal age, place of residence, education, or income is also noteworthy. It may suggest that, in this setting, more proximal lived conditions such as workload, partner support, and reproductive experiences are more influential for stress exposure than socioeconomic indicators alone. This interpretation is consistent with growing literature emphasizing that the timing, chronicity, and contextual meaning of stress may be more informative than simple demographic categories [22,23,24]. Prior literature likewise suggests that associations between stress and urbanicity or education are often inconsistent and highly context-dependent [2,16].

Occupational factors showed clear associations with stress. Women in the study group were more often employed, more likely to work overtime during pregnancy, and more likely to continue their usual workload without adjustment. These findings suggest that workplace demands may substantially contribute to prenatal stress, particularly when pregnancy is not accompanied by workload modification or supportive occupational policies. This interpretation is consistent with evidence that employment-related screening in pregnancy often fails to capture the working conditions most relevant to maternal stress [3].

Social context was also important. Women with higher stress were more likely to live alone or with fewer household members and were slightly less likely to report having a partner. These findings suggest that limited household and partner support may increase vulnerability to prenatal stress by reducing emotional reassurance and practical assistance during pregnancy [25,26]. Similarly, a history of spontaneous and induced abortion was more common among stress-exposed women. Although causal direction cannot be inferred, these findings are consistent with evidence suggesting bidirectional links between psychological distress and adverse reproductive experiences; previous reproductive loss may heighten anxiety in a subsequent pregnancy, while chronic stress may also contribute to reproductive complications through neuroendocrine and inflammatory pathways [16,27,28].

The findings also suggest that maternal education may function as a protective social determinant. Associations with child outcomes appeared time-dependent, with selected advantages emerging in anthropometric and developmental indicators during infancy among children of more highly educated mothers. Maternal education likely operates through cumulative pathways, including improved health literacy, use of antenatal and child health services, feeding practices, and enriched caregiving environments [20,29,30].

Overall, the findings support growing evidence that prenatal maternal stress is an important determinant of early child health and development and suggest that integrating psychosocial assessment, maternal support, and early developmental monitoring into routine antenatal and paediatric care may help reduce developmental disparities from the earliest stages of life.

## 5. Conclusions

Prenatal maternal stress was associated with poorer early physical growth and less favorable neurodevelopmental outcomes during the first year of life. These findings highlight the importance of integrating psychosocial support and early developmental monitoring into maternal and child healthcare.

### 5.1. Global Health and Policy Implications

The findings have important implications for global maternal and child health, particularly within the framework of early childhood development as a determinant of population health and human capital [19,20,30]. Prenatal psychological stress represents a widespread yet insufficiently addressed risk factor, particularly in populations affected by poverty, conflict, displacement, and social instability. The persistence of developmental differences across infancy suggests that early neurodevelopmental risk may accumulate over time, contributing to later inequalities in education, productivity, and health [2,16,20,29,30]. These findings align with WHO priorities and Sustainable Development Goal 3, including targets related to maternal health, child development, and mental health [12,13,14], and are also consistent with the WHO-UNICEF-World Bank Nurturing Care Framework [19,21].

This study provides novel evidence from Georgia, a post-Soviet context underrepresented in the global literature. By demonstrating concordance between self-reported stress and biological markers, and linking stress exposure to measurable infant outcomes, it contributes to the growing understanding of prenatal stress as a determinant of child health. From a public health perspective, the results support psychosocial risk assessment during antenatal care, targeted support for women with occupational strain, limited social support, or prior reproductive loss, and early developmental surveillance of infants at risk [3,19,21]. Parent-completed developmental screening tools such as the ASQ-3 may offer a low-cost, scalable, and culturally adaptable strategy for early identification and timely intervention [29,30]. Such approaches may help reduce developmental disparities and interrupt intergenerational cycles of health inequity [19,20,29,30].

### 5.2. Strengths and Limitations

This study has several strengths, including its longitudinal design, repeated assessments at standardized developmental stages, and use of a widely validated screening instrument administered with paediatrician support [31].

Several limitations should also be acknowledged.

Although the initial study cohort consisted of 398 pregnant women, the longitudinal and cortisol-related analyses were conducted in a predefined randomly selected subgroup of 190 mother–infant dyads (95 participants from each study group). This reduction in sample size was determined by the study design and did not result from participant attrition or loss to follow-up. Nevertheless, analyses performed in the subgroup may have reduced statistical power and may limit the generalizability of the longitudinal findings to the full study population.

Because salivary cortisol was measured only in the stress-exposed subgroup, direct comparisons of cortisol concentrations between stressed and control participants were not possible. Future studies should include biological stress measurements in both groups to enable comprehensive assessment of physiological differences associated with prenatal stress.

The ASQ-3 is a screening rather than diagnostic tool, and outcomes reflect developmental risk rather than confirmed developmental disorders [29,30]. Second, reliance on parent-reported data may have introduced reporting bias, although paediatric guidance during completion likely reduced this risk. Third, as an observational study, residual confounding by unmeasured postnatal environmental factors cannot be excluded.

An officially validated Georgian version of the ASQ-3 was unavailable at the time of the study. Although the instrument underwent a structured process of forward translation, back-translation, expert review, and pilot testing prior to use, formal psychometric validation of the Georgian adaptation, including assessments of internal consistency, test–retest reliability, construct validity, and inter-rater reliability, was not performed. Therefore, some measurement uncertainty cannot be completely excluded. Future studies should formally evaluate the psychometric properties of the Georgian ASQ-3 in larger representative samples to further strengthen its application in developmental research and clinical practice.

Finally, the findings may not be directly generalizable to populations with substantially different socioeconomic or healthcare contexts. Future longitudinal studies should examine whether these early developmental differences persist into later childhood and evaluate whether integrated maternal mental health and early childhood interventions can modify developmental trajectories [13,14,16,19].

## Figures and Tables

**Table 1:** Baseline Characteristics of the women participating in the study are presented in this table, considering gestational age, age, residency, health status, willingness to participate, access to prenatal care.

Baseline Characteristics of the Study Participants	
Characteristics	Definitions
**Gestational age**	Pregnant women in their first, second or third trimester were included to focus on different stages of pregnancy
**Age**	Women aged 18 years and older were eligible to ensure that the participants were legally able to consent and represent adult pregnancy experiences
**Residency**	Participants had to be residents of the city Tbilisi, Georgia, to maintain a consistent cultural and socio-economic context for the study
**Health status**	Women with no pre-existing psychological disorders or severe medical conditions (any chronic diseases) that could independently affect stress levels or cortisol concentrations were included to avoid confounding factors
**Willingness to participate**	Only women who provided informed consent and agreed to complete the pregnancy questionnaires and to give saliva samples for saliva cortisol concentration measurement were included
**Access to Prenatal Care**	Participants were required to be performing regular visits to the gynecologists, receiving regular prenatal care

## Data Availability

All relevant data are presented in the article. The study’s data cannot be publicly shared due to ethical restrictions, i.e., the data availability would compromise participant confidentiality. All relevant data are available upon request. Interested researchers may contact the author, N. Masiukovichi (nia_masiukovich@yahoo.com).

## Supplementary Materials

The following supporting information can be downloaded at: https://www.mdpi.com/article/10.3390/healthcare14152247/s1, Supplementary Figure S1: Factors Associated With Stress During Pregnancy: Comparison Of Study (Group 1) And Control (Group 2) Groups. Supplementary Figure S2: Comparison Of the Study And Control Groups According To The Delivery Complicatrions. Supplementary Figure S3: Comparison Of The Weakness Of the Delivery Activities Between The Study And Control Groups. Supplementary Figure S4: Comparison of 1-Minute APGAR Scores Between Study and Control Groups. Supplementary Figure S5: ASQ-3 Identified Anxiety In The Study And Control Groups. Supplementary Figure S6: Saliva Cortisol Levels in GROUP 1 (N = 95 randomly performed out of N = 172). Supplementary Table S1: Occupational Characteristics During Pregnancy According to Study Group. Supplementary Table S2: Low ASQ–3 Gross Motor Scores in Children in the Study and Control Groups. supplementary Table S3: The percentages of low ASQ–3 Fine Motor scores in children in different age-groups in the study and control groups. Supplementary Table S4: Socioeconomic and Cultural Factors Associated with Stress During Pregnancy. Supplementary Table S5: Anthropometric Outcomes Across the First Year of Life: Comparison of Study and Control Groups. Supplementary Table S6. Anthropometric Parameter Gains Across the First Year of Life: Comparison of Study (N = 95) and Control (N = 95) Groups. Supplementary Table S7. Prevalence of Developmental Delays (Low ASQ-3 Scores) Across Domains: Comparison of Study and Control Groups. Supplementary Table S8: Body Weight Measurements During the First Year of Life: Comparison of Study and Control Groups. Supplementary Table S9. Prevalence of Low Body Weight During the First Year of Life: Comparison of Study and Control Groups. Supplementary Table S10: Body Length/Height Measurements During the First Year of Life: Comparison of Study and Control Groups. Supplementary Table S11: Head Circumference Measurements During the First Year of Life: Comparison of Study and Control Groups. Supplementary Table S12: Chest Circumference Measurements During the First Year of Life: Comparison of Study and Control Groups.
