# Supplementary material for: Prenatal Maternal Stress, Socioeconomic and Cultural Determinants, and Early Childhood Physical and Neurodevelopmental Outcomes: Evidence from a Prospective Cohort Study in Georgia
Source: Healthcare (Basel). 2026 Jul 23;14(15):2247. doi: 10.3390/healthcare14152247 (PMC13465513; doi:10.3390/healthcare14152247)
Supplement: Supplementary file 1 [file healthcare-14-02247-s001.zip › Supplementary Figure S1.pdf]

Supplementary Figure S1.

Factors Associated With Stress During Pregnancy: Comparison Of Study (Group 1) And Control (Group 2) Groups

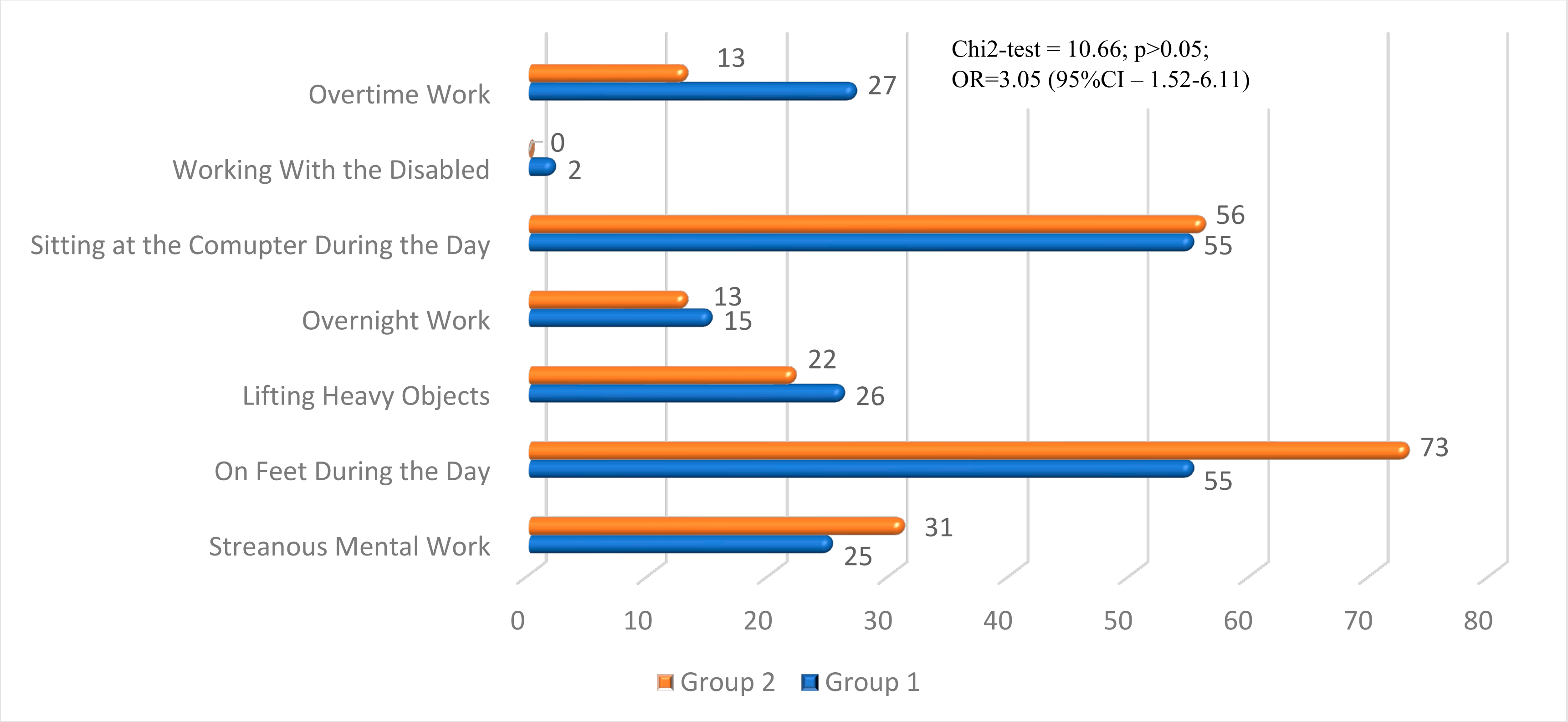

Notes: The figure compares selected occupational factors during pregnancy between the study and control groups. Overtime work was more frequent in the study group than in the control group (27 vs. 13), and the result was statistically significant (p>0.05). By contrast, prolonged standing during the day was reported more often in the control group (73 vs. 55), as was strenuous mental work (31 vs. 25). Sitting at the computer during the day was similarly common in both groups (55 vs. 56). Overall, the figure suggests differences in the pattern of occupational exposures during pregnancy between the two groups, particularly with regard to overtime work and physical workload.
