# Supplementary material for: Prenatal Maternal Stress, Socioeconomic and Cultural Determinants, and Early Childhood Physical and Neurodevelopmental Outcomes: Evidence from a Prospective Cohort Study in Georgia
Source: Healthcare (Basel). 2026 Jul 23;14(15):2247. doi: 10.3390/healthcare14152247 (PMC13465513; doi:10.3390/healthcare14152247)
Supplement: Supplementary file 1 [file healthcare-14-02247-s001.zip › Supplementary Figure S2.pdf]

Supplementary Figure S2.

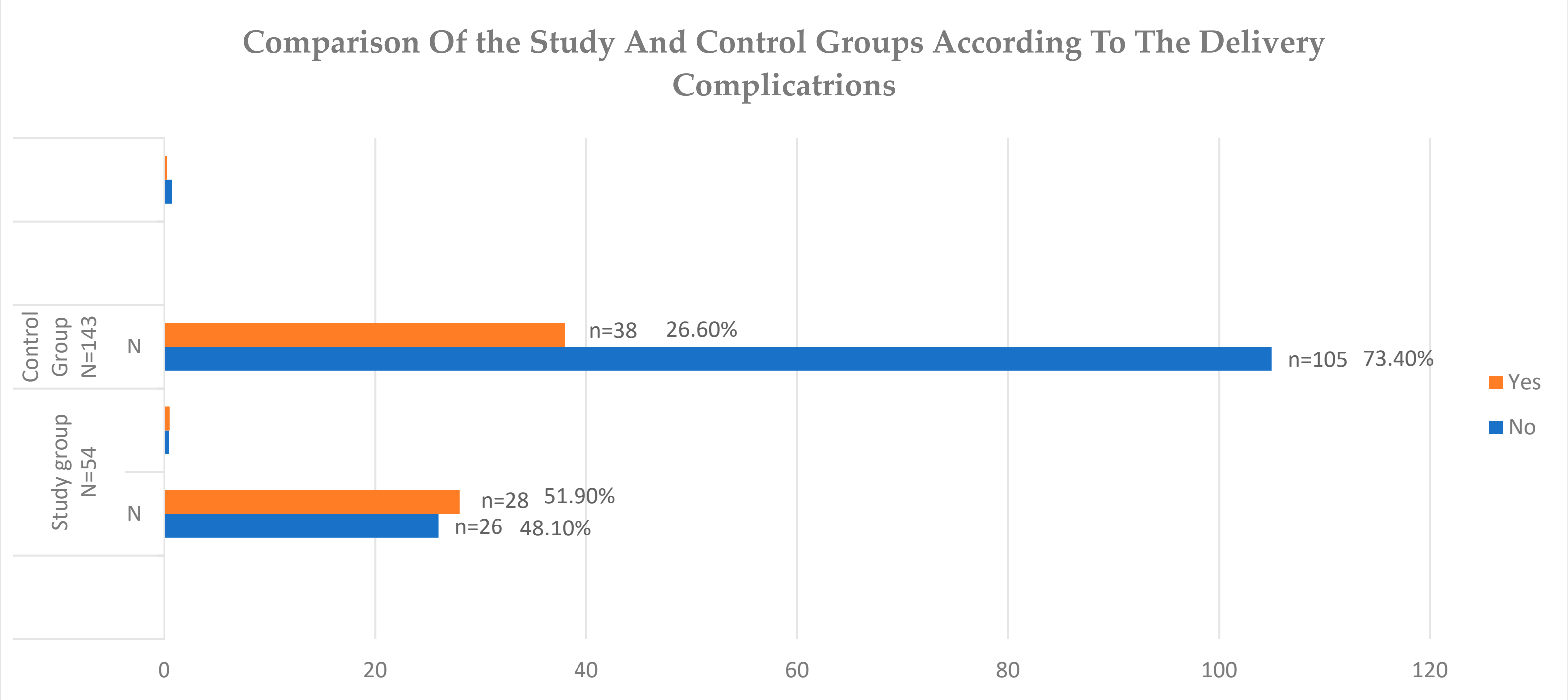

OR (95%Ci) 2.98 (1.55-5.70), p=0.001

Notes: Delivery complications were significantly more frequent in the study group than in the control group. The odds of delivery complications were nearly three times higher in the study group (OR = 2.98; 95% CI: 1.55–5.70; p = 0.001). Abbreviations: OR, odds ratio; CI, confidence interval.
