# Supplementary material for: Prenatal Maternal Stress, Socioeconomic and Cultural Determinants, and Early Childhood Physical and Neurodevelopmental Outcomes: Evidence from a Prospective Cohort Study in Georgia
Source: Healthcare (Basel). 2026 Jul 23;14(15):2247. doi: 10.3390/healthcare14152247 (PMC13465513; doi:10.3390/healthcare14152247)
Supplement: Supplementary file 1 [file healthcare-14-02247-s001.zip › Supplementary Figure S3.pdf]

Supplementary Figure S3.

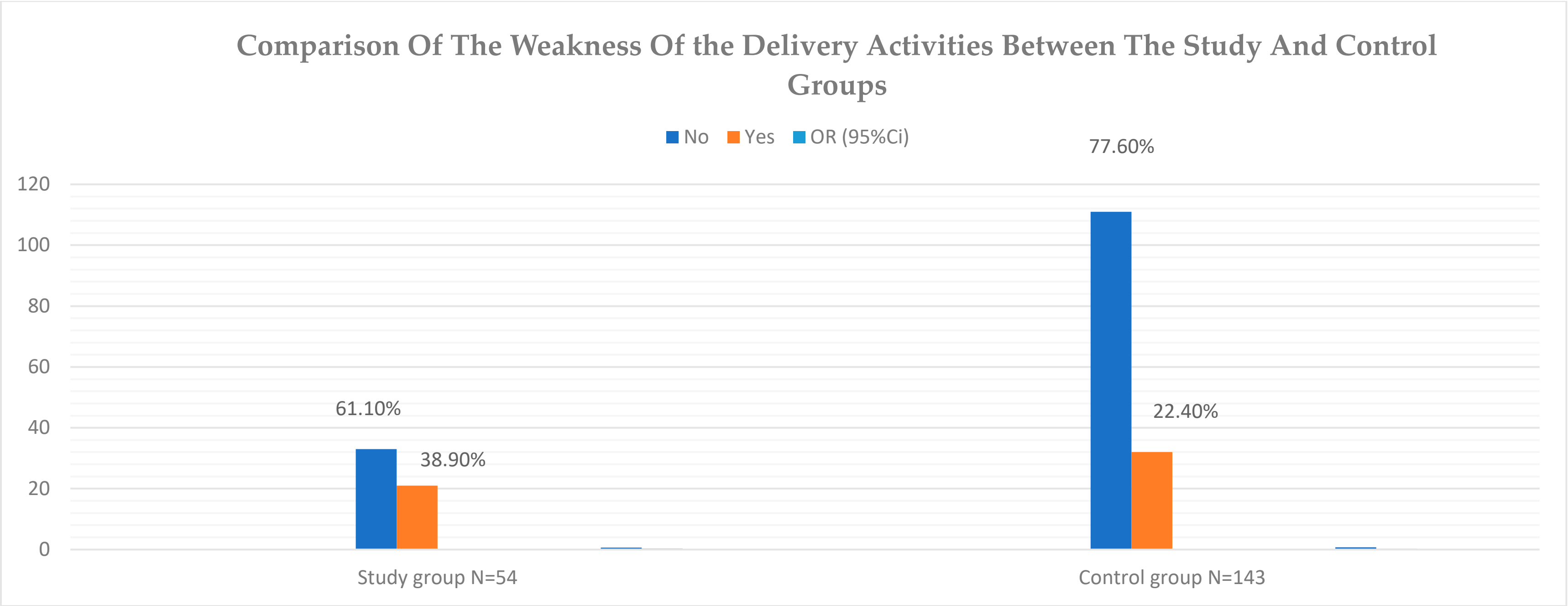

OR (95%CI) 2.21 (1.13-4.33), p=0.021

Notes: Weakness of labor activity was significantly more frequent in the study group than in the control group. Women in the study group had more than twofold higher odds of this complication compared with controls (OR = 2.21; 95% CI: 1.13–4.33; p = 0.021). Data are presented as proportions within each group. Between-group comparison was performed using the chi-square test.
