# Supplementary material for: Prenatal Maternal Stress, Socioeconomic and Cultural Determinants, and Early Childhood Physical and Neurodevelopmental Outcomes: Evidence from a Prospective Cohort Study in Georgia
Source: Healthcare (Basel). 2026 Jul 23;14(15):2247. doi: 10.3390/healthcare14152247 (PMC13465513; doi:10.3390/healthcare14152247)
Supplement: Supplementary file 1 [file healthcare-14-02247-s001.zip › Supplementary Figure S4.pdf]

Supplementary Figure S4.

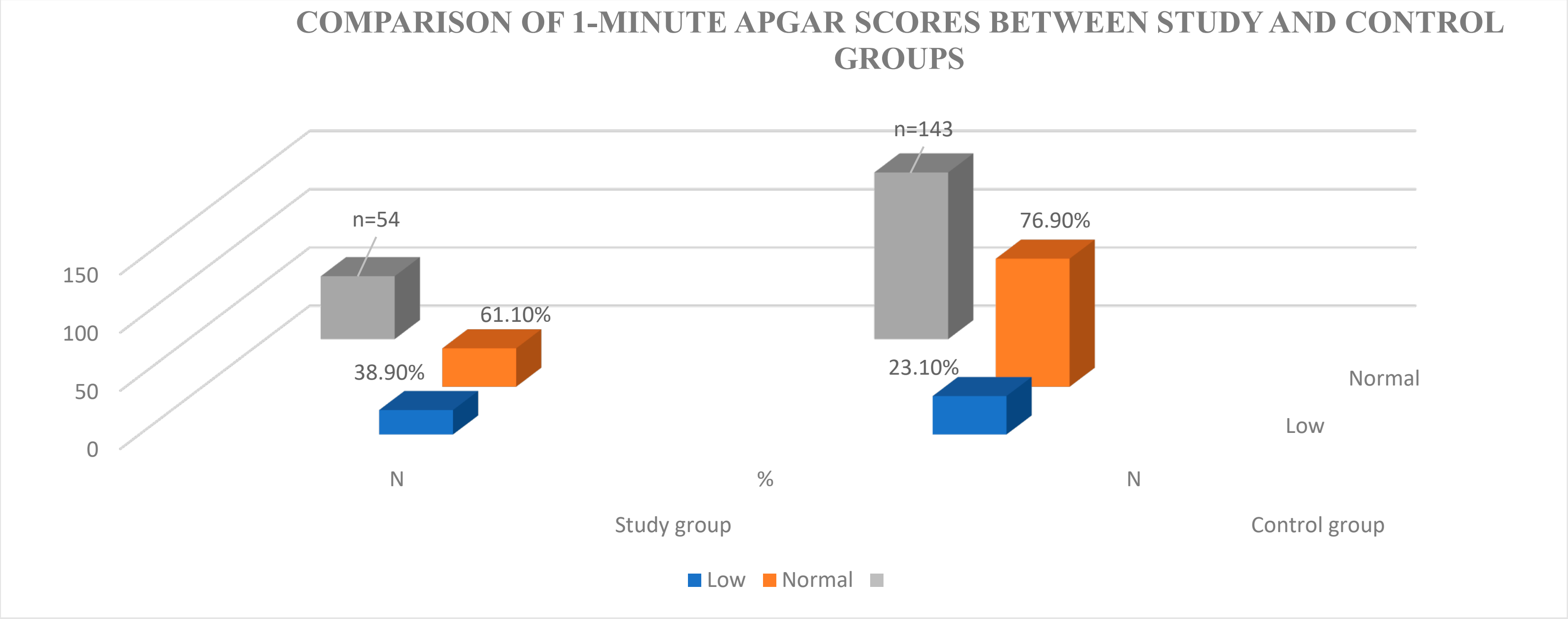

OR (95%CI) 2.12, p=0.028

Notes: Low 1-minute APGAR scores were more frequent in the study group than in the control group. Infants in the study group had significantly higher odds of a low 1-minute APGAR score compared with controls (OR = 2.12; p = 0.028), indicating a less favorable immediate neonatal condition in the study group.
