# Supplementary material for: Prenatal Maternal Stress, Socioeconomic and Cultural Determinants, and Early Childhood Physical and Neurodevelopmental Outcomes: Evidence from a Prospective Cohort Study in Georgia
Source: Healthcare (Basel). 2026 Jul 23;14(15):2247. doi: 10.3390/healthcare14152247 (PMC13465513; doi:10.3390/healthcare14152247)
Supplement: Supplementary file 1 [file healthcare-14-02247-s001.zip › Supplementary Figure S5.pdf]

Supplementary Figure S5.

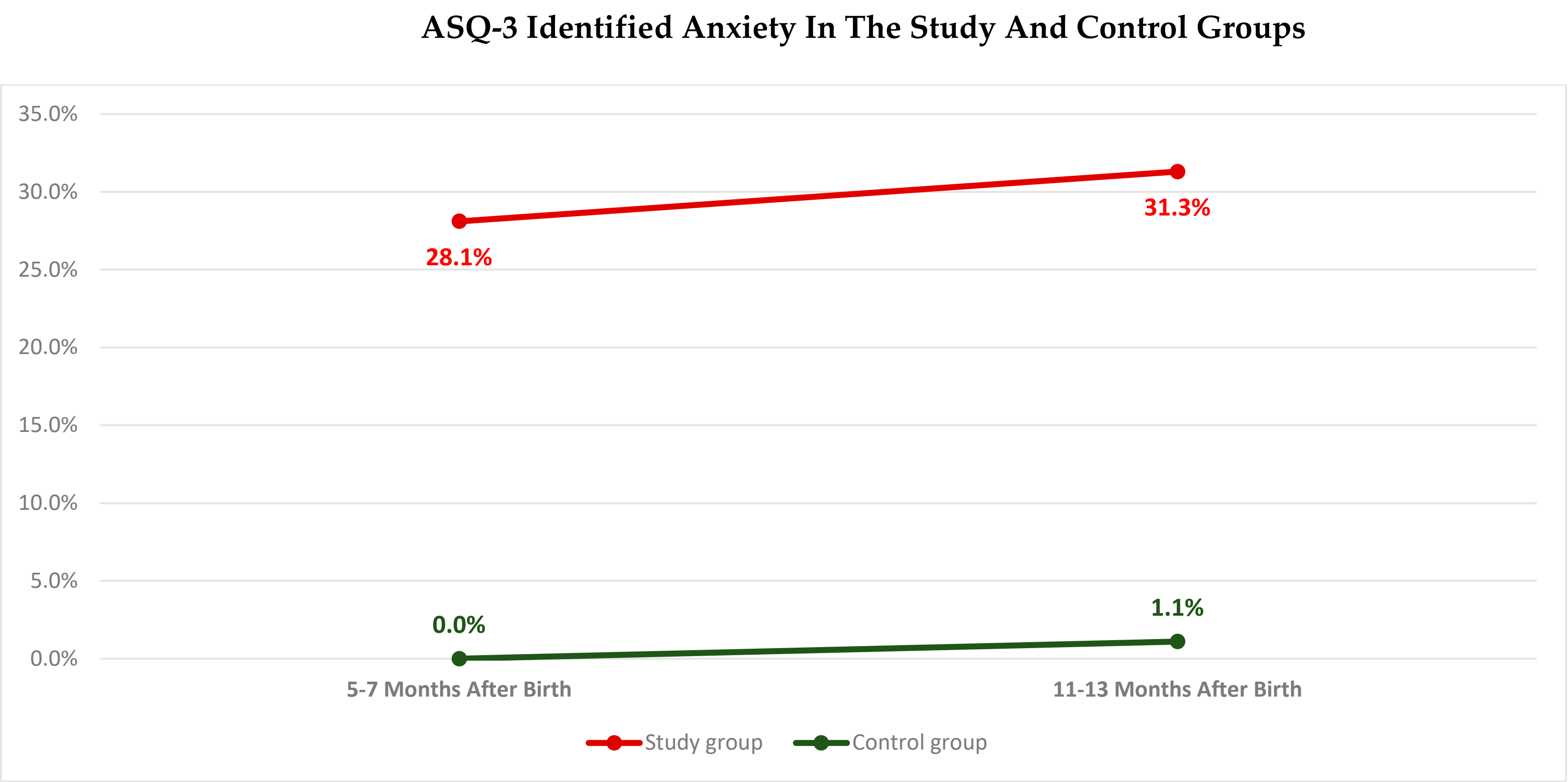

Notes: Anxiety-related concerns identified using the ASQ-3 were significantly more frequent in the study group than in the control group at both follow-up assessments. At 5–7 months after birth, anxiety-related concerns were observed in 28.1% of infants in the study group and in 0.0% of infants in the control group ($\chi^2 = 31.31$, $p < 0.001$). At 11–13 months after birth, the prevalence was 31.3% in the study group compared with 1.1% in the control group ($\chi^2 = 32.25$, $p < 0.001$).
