# Supplementary material for: Prenatal Maternal Stress, Socioeconomic and Cultural Determinants, and Early Childhood Physical and Neurodevelopmental Outcomes: Evidence from a Prospective Cohort Study in Georgia
Source: Healthcare (Basel). 2026 Jul 23;14(15):2247. doi: 10.3390/healthcare14152247 (PMC13465513; doi:10.3390/healthcare14152247)
Supplement: Supplementary file 1 [file healthcare-14-02247-s001.zip › Supplementary Figure S6.pdf]

Supplementary Figure S6.

Saliva Cortisol Levels in GROUP 1 (N=95 randomly performed out of N=172)

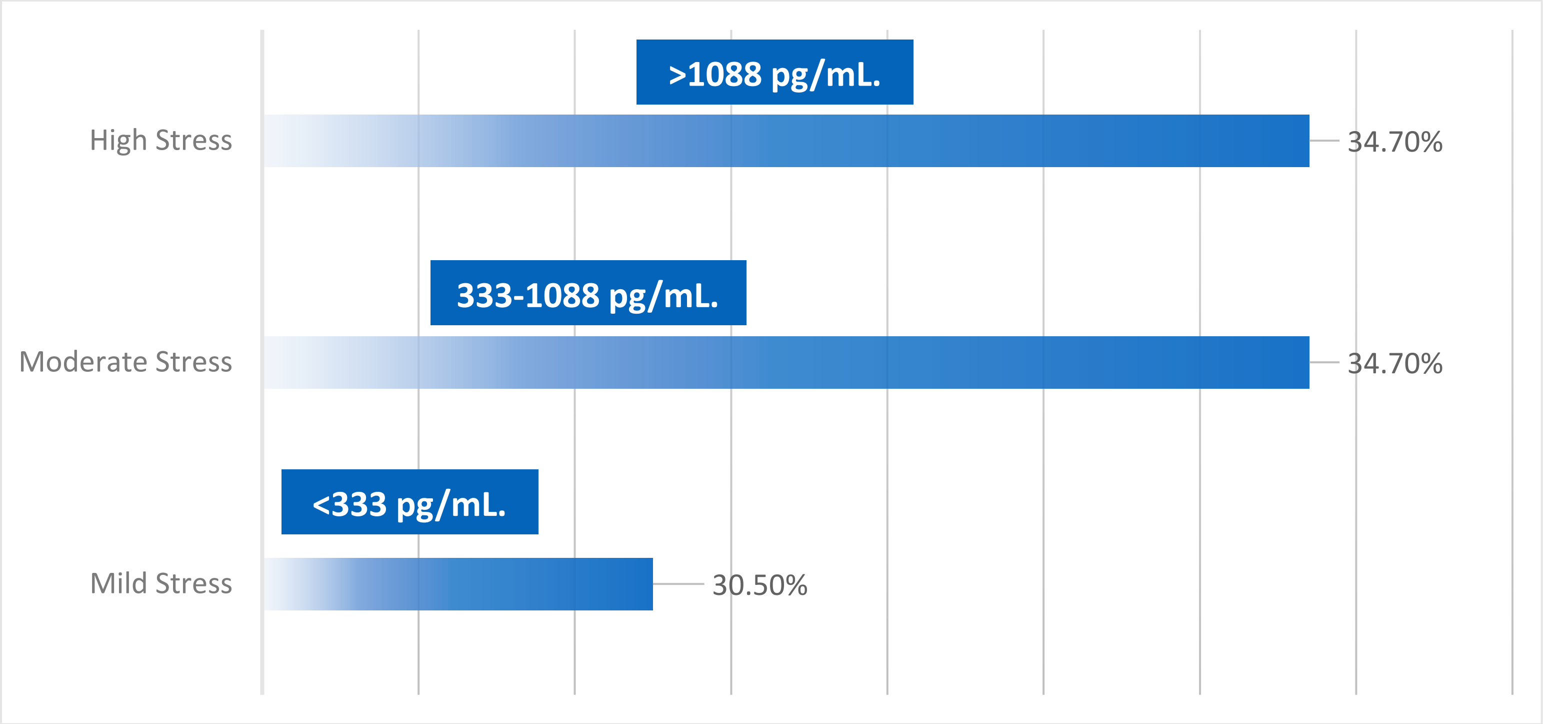

Mild stress (saliva cortisol concentration < 333 pg/ml) – n=29 (30.5%);
Moderate stress (saliva cortisol concentration 333-1088 pg/ml) – n=33 (34.7%);
High stress (saliva cortisol concentration >1088 pg/ml) – n=33 (34.7%).

Notes: Salivary cortisol concentrations were assessed in a randomly selected subsample of women from the study group (n = 95). Mild stress was defined as a cortisol concentration <333 pg/mL and was observed in 29 women (30.5%). Moderate stress (333–1088 pg/mL) and high stress (>1088 pg/mL) were each observed in 33 women (34.7%). Overall, 69.4% of the assessed women had moderate-to-high salivary cortisol levels, indicating elevated biological stress within this subgroup.

Abbreviation: pg/mL, picograms per milliliter.
