# Supplementary material for: Prenatal Maternal Stress, Socioeconomic and Cultural Determinants, and Early Childhood Physical and Neurodevelopmental Outcomes: Evidence from a Prospective Cohort Study in Georgia
Source: Healthcare (Basel). 2026 Jul 23;14(15):2247. doi: 10.3390/healthcare14152247 (PMC13465513; doi:10.3390/healthcare14152247)
Supplement: Supplementary file 1 [file healthcare-14-02247-s001.zip › Supplementary Table S1.pdf]

Supplementary Table S1.

Occupational Characteristics During Pregnancy According to Study Group

Occupational Characteristic	Group 1 (n = 172), n (%)	Group 2 (n = 226), n (%)	χ^2	p-value	OR (95% CI)
Strenuous mental work	25 (14.5)	31 (13.7)	0.05	0.816	—
Standing/on feet during the day	55 (32.0)	73 (32.3)	0.004	0.945	—
Lifting heavy objects	26 (15.1)	22 (9.7)	2.66	0.103	—
Overnight work	15 (8.7)	13 (5.8)	0.22	0.641	—
Sitting at a computer during the day	55 (32.0)	56 (24.8)	2.51	0.113	—
Working with individuals with disabilities or in a hospital setting	2 (1.2)	0 (0.0)	2.63	0.105	—
**Overtime work**	**27 (15.7)**	**13 (5.8)**	**10.66**	**0.001**	**3.05 (1.52–6.11)**

***Statistically significant. Abbreviations:** *OR, odds ratio; CI, confidence interval.*

Notes: Data are presented as n (%). Between-group comparisons were performed using the χ^2 test. Overtime work was significantly more frequent among women in the study group than in the control group (OR = 3.05; 95% CI: 1.52–6.11; p = 0.001). No statistically significant differences were observed for the remaining occupational characteristics.
