# Supplementary material for: Prenatal Maternal Stress, Socioeconomic and Cultural Determinants, and Early Childhood Physical and Neurodevelopmental Outcomes: Evidence from a Prospective Cohort Study in Georgia
Source: Healthcare (Basel). 2026 Jul 23;14(15):2247. doi: 10.3390/healthcare14152247 (PMC13465513; doi:10.3390/healthcare14152247)
Supplement: Supplementary file 1 [file healthcare-14-02247-s001.zip › Supplementary Table S10.pdf]

Supplementary Table S10.

Body Length/Height Measurements During the First Year of Life: Comparison of Study and Control Groups

Study Period	Study Group (n=95) Mean ± SD (cm)	Control Group (n=95) Mean ± SD (cm)	t-test, p-value
At birth	51.4 ± 2.3	52.8 ± 1.9	4.57, <0.001
1–3 months after birth	58.0 ± 2.8	60.5 ± 2.0	7.08, <0.001
5–7 months after birth	65.8 ± 2.2	68.4 ± 1.5	9.52, <0.001
11–13 months after birth	73.0 ± 1.9	76.5 ± 1.7	13.38, <0.001

Notes: Data are presented as mean ± standard deviation (SD). Between-group comparisons were performed using independent-samples t-tests. Mean body length/height was significantly lower in the study group than in the control group at birth and throughout the first year of life (all $p < 0.001$).
