# Supplementary material for: Prenatal Maternal Stress, Socioeconomic and Cultural Determinants, and Early Childhood Physical and Neurodevelopmental Outcomes: Evidence from a Prospective Cohort Study in Georgia
Source: Healthcare (Basel). 2026 Jul 23;14(15):2247. doi: 10.3390/healthcare14152247 (PMC13465513; doi:10.3390/healthcare14152247)
Supplement: Supplementary file 1 [file healthcare-14-02247-s001.zip › Supplementary Table S11.pdf]

**Supplementary Table S11.**

## Head Circumference Measurements During the First Year of Life: Comparison of Study and Control Groups

| Study Period | Study Group (n=95) Mean ± SD (cm) | Control Group (n=95) Mean ± SD (cm) | t-test, p-value |
|---|---|---|---|
| At birth | 33.2 ± 2.0 | 34.3 ± 1.1 | 4.70, <0.001 |
| 1–3 months after birth | 38.3 ± 1.6 | 39.4 ± 1.1 | 5.52, <0.001 |
| 5–7 months after birth | 42.8 ± 1.7 | 44.4 ± 0.8 | 8.30, <0.001 |
| 11–13 months after birth | 45.6 ± 1.5 | 47.0 ± 0.8 | 8.03, <0.001 |

Notes: Data are presented as mean ± standard deviation (SD). Between-group comparisons were performed using independent-samples t-tests. Mean head circumference was significantly lower in the study group than in the control group at birth and throughout the first year of life (all $p < 0.001$).
