# Supplementary material for: Prenatal Maternal Stress, Socioeconomic and Cultural Determinants, and Early Childhood Physical and Neurodevelopmental Outcomes: Evidence from a Prospective Cohort Study in Georgia
Source: Healthcare (Basel). 2026 Jul 23;14(15):2247. doi: 10.3390/healthcare14152247 (PMC13465513; doi:10.3390/healthcare14152247)
Supplement: Supplementary file 1 [file healthcare-14-02247-s001.zip › Supplementary Table S12.pdf]

Supplementary Table S12.

Chest Circumference Measurements During the First Year of Life: Comparison of Study and Control Groups

Study Period	Study Group (n=95) Mean ± SD (cm)	Control Group (n=95) Mean ± SD (cm)	t-test, p-value
At birth	31.5 ± 2.0	32.8 ± 1.1	5.55, <0.001
1–3 months after birth	35.6 ± 1.9	37.1 ± 1.4	6.20, <0.001
5–7 months after birth	41.8 ± 1.7	43.2 ± 0.9	7.09, <0.001
11–13 months after birth	47.3 ± 1.6	48.9 ± 1.0	8.27, <0.001

Notes: Data are presented as mean ± standard deviation (SD). Between-group comparisons were performed using independent-samples t-tests. Mean chest circumference was significantly lower in the study group than in the control group at birth and throughout the first year of life (all $p < 0.001$).
