# Supplementary material for: Prenatal Maternal Stress, Socioeconomic and Cultural Determinants, and Early Childhood Physical and Neurodevelopmental Outcomes: Evidence from a Prospective Cohort Study in Georgia
Source: Healthcare (Basel). 2026 Jul 23;14(15):2247. doi: 10.3390/healthcare14152247 (PMC13465513; doi:10.3390/healthcare14152247)
Supplement: Supplementary file 1 [file healthcare-14-02247-s001.zip › Supplementary Table S2.pdf]

Supplementary Table S2.

Low ASQ–3 Gross Motor Scores in Children in the Study and Control Groups

Assessment Stage	Study Group, n	Study Group, %	Control Group, n	Control Group, %	χ^2, p-value
1–3 months after birth	51	53.7	28	29.5	11.40, <0.001
5–7 months after birth	22	23.2	0	0.0	24.75, <0.001
11–13 months after birth	18	18.9	0	0.0	19.78, <0.001

Notes: Data are presented as n (%) of children with low ASQ-3 gross motor scores. Between-group comparisons were performed using the χ^2 test. Statistically significant differences were observed at all assessment stages ($p < 0.001$), with a higher prevalence of low gross motor scores in the study group compared with the control group.
