# Supplementary material for: Prenatal Maternal Stress, Socioeconomic and Cultural Determinants, and Early Childhood Physical and Neurodevelopmental Outcomes: Evidence from a Prospective Cohort Study in Georgia
Source: Healthcare (Basel). 2026 Jul 23;14(15):2247. doi: 10.3390/healthcare14152247 (PMC13465513; doi:10.3390/healthcare14152247)
Supplement: Supplementary file 1 [file healthcare-14-02247-s001.zip › supplementary Table S3.pdf]

Supplementary Table S3.

The percentages of low ASQ–3 Fine Motor scores in children in different age-groups in the study and control groups

Assessment Stage	Study Group, n	Study Group, %	Control Group, n	Control Group, %	χ^2, p-value
1–3 months after birth	58	61.1	12	12.6	47.61, <0.001
5–7 months after birth	47	49.5	0	0.0	62.12, <0.001
11–13 months after birth	36	37.9	0	0.0	44.18, <0.001

Notes: Data are presented as n (%). Between-group comparisons were performed using the χ^2 test. Low ASQ-3 fine motor scores were significantly more frequent in the study group than in the control group at all postnatal assessment stages (all $p < 0.001$).
