# Supplementary material for: Prenatal Maternal Stress, Socioeconomic and Cultural Determinants, and Early Childhood Physical and Neurodevelopmental Outcomes: Evidence from a Prospective Cohort Study in Georgia
Source: Healthcare (Basel). 2026 Jul 23;14(15):2247. doi: 10.3390/healthcare14152247 (PMC13465513; doi:10.3390/healthcare14152247)
Supplement: Supplementary file 1 [file healthcare-14-02247-s001.zip › Supplementary Table S4.pdf]

Supplementary Table S4.

Socioeconomic and Cultural Factors Associated with Stress During Pregnancy

Variable	Study Group (n=172), %	Control Group (n=226), %	OR (95% CI)	p-value
Flat area <50 m²	22.9	15.0	1.67 (0.70–3.99)	χ^2=77.75; <0.001
Flat area 51–100 m²	40.0	49.1	1.45 (0.70–2.99)	χ^2=77.75; <0.001
Flat area 101–130 m²	22.9	17.3	1.42 (0.60–3.36)	χ^2=77.75; <0.001
Flat area 131–150 m²	11.4	15.5	1.42 (0.47–4.27)	χ^2=77.75; <0.001
Flat area >150 m²	2.9	3.1	1.09 (0.13–9.11)	χ^2=77.75; <0.001
Married/Has a partner	97.10	100	—	χ^2=6.64; p=0.01
Not married/divorced	2.9	0.0	—	χ^2=6.64; p=0.01
Temporarily unemployed	40.7	52.2	1.59 (1.07–2.38)	
Employed during pregnancy	59.3	47.8	1.59 (1.07–2.38)	0.019
Not working	75.6	85	1.82 (1.10–3.02)	0.019
Continuing usual work during pregnancy	24.2	15.0	1.82 (1.10–3.02)	0.019
Overtime work	17.1	5.8	3.39 (1.20–9.61)	0.016
History of spontaneous abortion	37.1	17.7	2.75 (1.28–5.91)	0.008
Living with 1–2 people	34.3	24.8	1.59 (1.02–2.45)	0.038
Living with 3+ people	65.70	75.20	1.59 (1.02–2.45)	0.038

Notes: Data are presented as percentages. Odds ratios (ORs) with 95% confidence intervals (CIs) were calculated to estimate associations with prenatal stress. Between-group comparisons were performed using χ^2 tests. Statistically significant associations ($p < 0.05$) were observed for marital status, employment, continuation of work during pregnancy, overtime work, previous spontaneous abortion, and household size. Although the overall distribution of residential area differed between groups, individual category-specific ORs were not statistically significant.
