# Supplementary material for: Prenatal Maternal Stress, Socioeconomic and Cultural Determinants, and Early Childhood Physical and Neurodevelopmental Outcomes: Evidence from a Prospective Cohort Study in Georgia
Source: Healthcare (Basel). 2026 Jul 23;14(15):2247. doi: 10.3390/healthcare14152247 (PMC13465513; doi:10.3390/healthcare14152247)
Supplement: Supplementary file 1 [file healthcare-14-02247-s001.zip › Supplementary Table S5.pdf]

Supplementary Table S5.

Anthropometric Outcomes Across the First Year of Life: Comparison of Study and Control Groups

Variable	Study Birth Mean±SD	Study 2 mo Mean±SD	Study 6 mo Mean±SD	Study 12 mo Mean±SD	Control Birth Mean±SD	Control 2 mo Mean±SD	Control 6 mo Mean±SD	Control 12 mo Mean±SD	p-value
Weight (g)	3056.3±373.2	4258.2±729.6	6553.6±744.2	8627.4±738.2	3391.6±399.7	5340.1±635.6	7506.8±762.9	10295.8±970.6	<0.001 (all)
t-test	*6.58*	*10.9*	*8.72*	*6.58*	*10.9*	*8.72*			
Length/Height (cm)	51.4±2.3	58.0±2.8	65.8±2.2	73.0±1.9	52.8±1.9	60.5±2.0	68.4±1.5	76.5±1.7	<0.001 (all)
t-test	*4.57*	*7.08*	*9.52*	*4.57*	*7.08*	*9.52*			
Head Circumference (cm)	33.2±2.0	38.3±1.6	42.8±1.7	45.6±1.5	34.3±1.1	39.4±1.1	44.4±0.8	47.0±0.8	<0.001 (all)
t-test	*4.7*	*5.52*	*8.3*	*4.7*	*5.52*	*8.3*			
Chest Circumference (cm)	31.5±2.0	35.6±1.9	41.8±1.7	47.3±1.6	32.8±1.1	37.1±1.4	43.2±0.9	48.9±1.0	<0.001 (all)
t-test	*5.55*	*6.2*	*7.09*	*5.55*	*6.2*	*7.09*			

Notes: Data are presented as mean ± SD. Between-group comparisons were performed using independent-samples t-tests. Children in the study group exhibited significantly lower anthropometric measurements than controls at birth and throughout the first year of life for all variables (all $p < 0.001$).
