# Supplementary material for: Prenatal Maternal Stress, Socioeconomic and Cultural Determinants, and Early Childhood Physical and Neurodevelopmental Outcomes: Evidence from a Prospective Cohort Study in Georgia
Source: Healthcare (Basel). 2026 Jul 23;14(15):2247. doi: 10.3390/healthcare14152247 (PMC13465513; doi:10.3390/healthcare14152247)
Supplement: Supplementary file 1 [file healthcare-14-02247-s001.zip › Supplementary Table S6.pdf]

Supplementary Table S6.

Anthropometric Parameter Gains Across the First Year of Life: Comparison of Study (N=95) and Control (N=95) Groups

Variable	Study 1–3 Months Mean ± SD	Study 5–7 Months Mean ± SD	Study 11–13 Months Mean ± SD	Control 1–3 Months Mean ± SD	Control 5–7 Months Mean ± SD	Control 11–13 Months Mean ± SD	p-value
Weight Gain (g)	1202 ± 514.5	3497.4 ± 548.7	5571.1 ± 634.2	1948.5 ± 503.6	4115.2 ± 583.0	6904.3 ± 852.3	<0.001 (all)
t-test	*10.11*	*7.52*	*12.23*	*10.11*	*7.53*	*12.23*	
Length/Height Gain (cm)	6.6 ± 1.3	14.5 ± 2.1	21.7 ± 2.2	7.7 ± 2.0	15.6 ± 1.8	23.7 ± 2.1	<0.001 (all)
t-test	*4.5*	*3.88*	*6.41*	*4.5*	*3.88*	*6.41*	
Head Circumference Gain (cm)	6.8 ± 1.4	9.5 ± 1.6	12.3 ± 1.8	6.6 ± 1.1	10.0 ± 1.1	12.7 ± 1.3	0.275; 0.013; 0.081
t-test	*1.1*	*2.51*	*1.76*	*1.1*	*2.51*	*1.76*	
Chest Circumference Gain (cm)	4.1 ± 1.8	10.4 ± 1.6	15.9 ± 2.1	4.3 ± 1.2	10.4 ± 1.1	16.1 ± 1.5	0.369; 0.960; 0.451
t-test	*0.9*	*0.005*	*0.76*	*0.9*	*0.005*	*0.76*	

Notes: Data are presented as mean ± SD. Between-group comparisons were performed using independent-samples t-tests. Weight gain and length/height gain were significantly lower in the study group than in the control group at all time points (all $p < 0.001$). Head circumference gain differed significantly only at 5–7 months ($p = 0.013$), while chest circumference gain did not differ significantly between groups.
