# Supplementary material for: Prenatal Maternal Stress, Socioeconomic and Cultural Determinants, and Early Childhood Physical and Neurodevelopmental Outcomes: Evidence from a Prospective Cohort Study in Georgia
Source: Healthcare (Basel). 2026 Jul 23;14(15):2247. doi: 10.3390/healthcare14152247 (PMC13465513; doi:10.3390/healthcare14152247)
Supplement: Supplementary file 1 [file healthcare-14-02247-s001.zip › Supplementary Table S7.pdf]

Supplementary Table S7.

Prevalence of Developmental Delays (Low ASQ-3 Scores) Across Domains: Comparison of Study and Control Groups

ASQ-3 Domain	Study Group 1–3 mo n (%)	Study Group 5–7 mo n (%)	Study Group 11–13 mo n (%)	Control Group 1–3 mo n (%)	Control Group 5–7 mo n (%)	Control Group 11–13 mo n (%)	p-value
Gross Motor	51 (53.7)	22 (23.2)	18 (18.9)	28 (29.5)	0 (0.0)	0 (0.0)	<0.001
χ^2	*11.4*	*24.75*	*19.78*	*11.4*	*24.75*	*19.78*	
Fine Motor	58 (61.1)	47 (49.5)	36 (37.9)	12 (12.6)	0 (0.0)	0 (0.0)	<0.001
χ^2	*47.61*	*62.12*	*44.18*	*47.61*	*62.12*	*44.18*	
Communication	69 (72.6)	78 (82.1)	75 (78.9)	28 (29.5)	7 (7.4)	7 (7.4)	<0.001
χ^2	*35.21*	*106.75*	*98.68*	*35.21*	*106.75*	*98.68*	
Problem Solving	76 (80.0)	91 (95.8)	91 (95.8)	26 (27.4)	4 (4.2)	4 (4.2)	<0.001
χ^2	*52.64*	*158.51*	*158.51*	*52.64*	*158.51*	*158.51*	
Personal–Social	76 (80.0)	94 (98.9)	92 (96.8)	33 (34.7)	2 (2.1)	2 (2.1)	<0.001
χ^2	*39.58*	*177.27*	*169.65*	*39.58*	*177.27*	*169.65*	

Notes: Data are presented as n (%). Between-group comparisons were performed using χ^2 tests. Developmental delays (low ASQ-3 scores) were significantly more frequent in the study group than in the control group across all developmental domains and assessment stages (all $p < 0.001$).
