# Supplementary material for: Prenatal Maternal Stress, Socioeconomic and Cultural Determinants, and Early Childhood Physical and Neurodevelopmental Outcomes: Evidence from a Prospective Cohort Study in Georgia
Source: Healthcare (Basel). 2026 Jul 23;14(15):2247. doi: 10.3390/healthcare14152247 (PMC13465513; doi:10.3390/healthcare14152247)
Supplement: Supplementary file 1 [file healthcare-14-02247-s001.zip › Supplementary Table S8.pdf]

Supplementary Table S8.

Body Weight Measurements During the First Year of Life: Comparison of Study and Control Groups

Study Period	Study Group (n=95) Mean ± SD (g)	Control Group (n=95) Mean ± SD (g)	t-test, p-value
At birth	3056.3 ± 373.2	3391.6 ± 399.7	6.58, <0.001
1–3 months after birth	4258.2 ± 729.6	5340.1 ± 635.6	10.90, <0.001
5–7 months after birth	6553.6 ± 744.2	7506.8 ± 762.9	8.72, <0.001
11–13 months after birth	8627.4 ± 738.2	10295.8 ± 970.6	13.34, <0.001

Notes: Data are presented as mean ± standard deviation (SD). Between-group comparisons were performed using independent-samples t-tests. Mean body weight was significantly lower in the study group than in the control group at birth and throughout the first year of life (all $p < 0.001$).
