# Supplementary material for: Prenatal Maternal Stress, Socioeconomic and Cultural Determinants, and Early Childhood Physical and Neurodevelopmental Outcomes: Evidence from a Prospective Cohort Study in Georgia
Source: Healthcare (Basel). 2026 Jul 23;14(15):2247. doi: 10.3390/healthcare14152247 (PMC13465513; doi:10.3390/healthcare14152247)
Supplement: Supplementary file 1 [file healthcare-14-02247-s001.zip › Supplementary Table S9.pdf]

Supplementary Table S9.

Prevalence of Low Body Weight During the First Year of Life: Comparison of Study and Control Groups

Assessment Stage	Study Group, n	Study Group, %	Control Group, n	Control Group, %	χ^2, p-value
At birth	5	5.0	0	0.0	5.11, 0.024
1–3 months after birth	48	50.5	0	0.0	63.89, <0.001
5–7 months after birth	29	30.5	7	6.9	16.50, <0.001
11–13 months after birth	13	13.7	7	6.9	2.00, 0.157

Notes: Data are presented as n (%). Between-group comparisons were performed using χ^2 tests. Low body weight was significantly more frequent in the study group at birth, 1–3 months, and 5–7 months. By 11–13 months, the difference between groups was no longer statistically significant ($p = 0.157$).
